# Is This Still Medical Education? The Educational Use of Nude Pediatric Clinical Photographs: Bioethical Reassessment

**DOI:** 10.2196/104023

**Published:** 2026-09-16

**Authors:** Iwona Bujek

**Affiliations:** 1 Poznan University of Medical Sciences Poznan, Greater Poland Poland; 2 GGZ Voor Elkaar, Mental Health Care Institution Utrecht, North Holland The Netherlands

**Keywords:** medical education, clinical photography, pediatric patients, bioethics, educational value, educational accountability, educational necessity, visual ethics, clinical images, medical publishing

## Abstract

Clinical photography can convey phenotypic, anatomical, and diagnostic information that may be difficult to replace with text. This also applies to highly sensitive pediatric clinical photographs when the scope of patient exposure is necessary to achieve a specific educational objective. This viewpoint addresses a more specific problem: “How should the educational function of a highly sensitive pediatric clinical photograph be evaluated when the condition identified with the photograph is substantively described elsewhere in the educational material?” The analysis is grounded in a documented longitudinal observation of 28 editions and reprints of a single academic textbook lineage published between 2005 and 2019, across which the same image-condition configuration persisted. At the point of image presentation, the depicted clinical conditions were explicitly identified, while the accompanying text stated that they were described elsewhere in the textbook, where their substantive clinical descriptions were located in accordance with the textbook’s declared curricular structure. On this basis, I propose educational disconnection to describe a situation in which the image-specific educational contribution at the point of presentation is difficult to reconstruct from the observable relationship among image location, the clinical condition identified with the image, the condition’s declared curricular location, the location of its substantive clinical description, and the destination indicated in the text accompanying the photograph. The central distinction is that identification of a clinical condition, identification of where that condition is substantively described, and identification of the educational contribution of its photograph are not the same. A separate question therefore remains: “What additional educational information does the image contribute here?” The longitudinal dimension extends this analysis through the concept of educational value decay, referring to the possibility that the relationship between an image and the context-specific justification for its presence may weaken over time, without assuming that educational value automatically declines with the age or repeated reproduction of the image. In response, I propose an Educational Reassessment Framework—a practical, event-triggered process that proceeds from identifying the image’s educational function and its relationship to the curricular structure, through assessment of its image-specific educational contribution and exposure-function proportionality, to the consideration of equivalent alternatives, the continuing validity of its justification, and the conditions of responsible continued use. Reassessment is not intended to automatically remove historical, repeatedly used, or highly sensitive photographs. Responsible reuse of an educationally valuable image may remain justified and may reduce the need to photograph additional patients, but educational value does not substitute for appropriate authorization or other conditions of responsible use. Its purpose is to determine whether continued use is supported by an identifiable, current, and context-specific educational contribution, whether the scope of patient information disclosed is necessary and proportionate to that contribution, and whether the conditions supporting use remain appropriate.

## Introduction

### Background

Clinical photography plays an important role in medical education. It can enable learners to recognize phenotypic features, anatomical changes, manifestations of disease, distributions of clinical features, and treatment outcomes that textual description alone may not convey with equal precision [1-6]. In certain circumstances, photographs depicting nudity, intimate anatomy, or other highly sensitive bodily features may likewise serve an important educational function that is difficult to replace [3,7-10]. If a particular anatomical feature, phenotype, or distribution of clinical findings is relevant to an educational objective, cropping or obscuring a clinically relevant area may reduce the educational information available from the image [1,3,11-14]. This viewpoint therefore does not challenge the use of such photographs as educational tools. The scope of patient exposure should, however, remain related to the information the image is expected to convey. Whole-body context may be necessary to assess phenotype, anatomical proportions, distribution of clinical features, or relationships among clinical characteristics [7,9,10]. The question is therefore not simply, “Could less be shown?” but rather “What scope of the image is necessary to convey the information required by the specific educational objective?”

A highly sensitive pediatric clinical photograph is a clinical photograph created during childhood that visually discloses nudity, intimate anatomy, sex-related anatomical characteristics, or other bodily features that reveal particularly sensitive information about the child.

This is a descriptive rather than normative category. It characterizes the nature and scope of the visual information disclosed and does not, by itself, imply that the photograph lacks educational value, is inappropriate, or should not be used. Its educational justification depends separately on the function the image serves and on whether the scope of the information disclosed is necessary and proportionate to that function.

This question should be distinguished from identifiability. Cropping, exposure minimization, and deidentification address related but nonidentical problems [1,15-17]. Removing or obscuring the face does not necessarily eliminate identification based on body shape, distinctive anatomical features, scars, birthmarks, a rare phenotype, or contextual information [1,15-18]. At the same time, restricting an image solely to reduce identifiability may remove clinically relevant information [1,3,15]. The scope of an image should therefore be evaluated both in terms of the information it reveals about the patient and the information it contributes to the educational objective, while maintaining appropriate conditions of consent, privacy, and protection against identification [1,15,19-22].

Repeated use of the same photograph does not in itself imply loss of educational value. Particularly for rare clinical conditions, a high-quality image may remain a useful educational resource for many years, and responsible reuse may reduce the need to photograph additional patients [1-3,23]. This potential advantage of reuse, however, does not substitute for appropriate authorization of the continued use of the existing image. Avoiding unnecessary photography of additional patients should not be achieved by concentrating the cumulative burden of repeated exposure on an individual who did not authorize that use. Responsible reuse should therefore protect both patients who might otherwise be newly photographed and the person whose existing image is repeatedly used. Continued use nevertheless requires two questions to be distinguished: whether the image continues to provide an identifiable educational contribution and whether its use remains supported by appropriate conditions of use [1,15,19-23].

These issues are particularly important for highly sensitive pediatric photographs, where potential educational benefit coexists with extensive disclosure of information about a person who, when the photograph was taken, had limited or no capacity to determine its future use [7-9,24-26].

The literature on clinical photography has focused primarily on consent, privacy, confidentiality, identifiability, deidentification, digital dissemination, patient perspectives on the acceptability and use of medical photographs, and protection of children and other groups requiring heightened safeguards [1,7,8,15,19-22,27-30].

These issues remain fundamental, but they do not exhaust a separate educational question: “What is the relationship between the scope of information disclosed by a highly sensitive image and its specific educational contribution at the point at which it is presented?”

This question arose from a documented longitudinal observation of a single academic textbook lineage. Across 28 documented editions and reprints published between 2005 and 2019, the same highly sensitive pediatric clinical photographs remained within a recurring image-condition configuration. At the point of image presentation, the depicted clinical conditions were explicitly identified, but were not identified in the textbook’s table of contents as components of the chapter containing the photographs. Instead, the accompanying text stated that the respective conditions were described elsewhere in the textbook, where their substantive clinical descriptions were located in accordance with the textbook’s declared curricular structure.

This produced an observable asymmetry: the declared curricular location of the condition, the location of its substantive clinical description, and the destination indicated in the text accompanying the photograph converged elsewhere, while the highly sensitive pediatric photograph remained at a different location. Its educational contribution at the point of presentation therefore cannot be derived solely from identification of the depicted condition, the location of its substantive clinical description, or the accompanying reference to that other location.

The central distinction of this viewpoint is that identification of the depicted clinical condition, identification of where that condition is substantively described, and identification of the educational contribution of its photograph are analytically distinct elements. A photograph may identify what condition is depicted; the textbook structure may indicate where that condition is addressed; and the accompanying text may state that the condition is described elsewhere. A separate question remains: “What additional educational information does the photograph itself contribute at the point of its presentation?”

This analysis neither assumes that learners use chapters in a particular sequence nor reconstructs the intentions, learning objectives, or educational reasoning of the author, editor, or publisher. Physical separation between a photograph and the substantive clinical description of the depicted condition is not inherently problematic; cross-referencing may be an appropriate component of educational design [11,12,31-35], and an image may serve a distinct educational function outside the location in which the condition is substantively described.

I refer to educational disconnection as a situation in which the image-specific educational contribution at the point of presentation remains difficult to reconstruct from the observable relationship among the photographs, the condition identified with it, the condition’s declared curricular location, the location of its substantive clinical description, and the destination indicated in the accompanying text. The term does not imply inappropriate use or absence of educational value; it signals that the image’s context-specific educational contribution requires clarification. Only after this contribution has been identified can exposure-function proportionality be meaningfully assessed.

The longitudinal dimension adds a second question: “If the same photograph remains within the same image-condition configuration across successive editions and reprints, does the context-specific justification for its presence at that particular location remain current?” I refer to the possibility that the relationship between an image and its context-specific educational justification may weaken over time as educational value decays. This does not assume that time or repetition automatically diminishes educational value.

The continuing validity of educational justification is distinct from the basis for continued use. An image may retain educational value while the conditions supporting later reuse require reassessment; conversely, an appropriate basis for use does not itself establish the educational necessity of the image at a particular location [1,15,19-23].

On this basis, this viewpoint proposes an Educational Reassessment Framework—a practical, event-triggered process that begins by asking what educational contribution the image provides at its point of presentation and how its location relates to the observable structure of the material. It then assesses image-specific educational contribution, exposure-function proportionality, available alternatives, continuing validity of justification, and conditions of responsible continued use. The educational question developed here is not format-specific and may arise across printed and digital textbooks, multimedia teaching materials, online educational resources, and undergraduate, postgraduate, or continuing professional education [36-39].

### Longitudinal Observation Underlying the Viewpoint

This viewpoint is neither a systematic literature review nor an empirical study designed to estimate the prevalence of the phenomenon under consideration in medical education. Its starting point is a documented longitudinal observation of specific educational material, used to identify and conceptualize an educational problem rather than to infer its prevalence.

The reconstruction began after an adult recognized, in 2023, clinical photographs taken of her during childhood that remained available in educational material. Using the name of the physician who had provided treatment during childhood as an identifying reference point, the earlier trajectory of image use was reconstructed from books and textbook publications, conference materials, ebooks, PDFs, presentations, and other available educational and digital resources. The broader reconstruction identified the same pediatric clinical photographs across more than 40 publications and educational or digital resources; this wider long-term reuse and governance trajectory has been reported previously [40].

For the purposes of the present viewpoint, the analysis was narrowed to a single academic textbook lineage , providing a bounded context in which the relationship between the photographs, the clinical conditions identified with them, and the location of their substantive clinical descriptions could be followed longitudinally. Twenty-eight documented editions and reprints containing the analyzed photograph were identified within this textbook lineage between 2005 and 2019. Continued curricular use of existing editions remains documented in 2026.

The more than 40 publications and educational or digital resources establish the wider dissemination trajectory, whereas the 28 documented editions and reprints constitute the bounded longitudinal sequence analyzed here. The present analysis therefore concerns the persistence of an observable image-condition configuration within this textbook lineage.

Across the analyzed sequence, several highly sensitive pediatric clinical photographs were presented with explicit identification of the depicted clinical conditions. Those conditions were not identified in the textbook’s table of contents as components of the chapter containing the photographs. Instead, the accompanying text stated that the respective conditions were described elsewhere in the textbook. Examination of those locations confirmed that their substantive clinical descriptions were provided there, consistent with the textbook’s declared curricular structure.

The observable image-condition configuration comprised five elements, which were (1) the clinical photograph ; (2) the clinical condition identified at the point of image presentation ; (3) the condition’s declared curricular location within the textbook structure; (4) the location of the condition’s substantive clinical description; and (5) the destination indicated in the text accompanying the photograph.

These elements did not converge on the same location.The condition was identified beside the photograph; its substantive clinical description and the destination indicated in the accompanying text were located elsewhere, consistent with its declared curricular location, while the highly sensitive pediatric clinical photograph remained at a different location.

This configuration persisted across the 28 editions and reprints identified between 2005 and 2019 . The central object of observation was therefore not the number of reproductions itself, but the persistence of this observable relationship across successive editions and reprints.

This observation neither permits determination of the intentions, learning objectives, or educational reasoning of the authors, editors, or publisher nor establishes that the photograph lacked educational value. Physical separation between a clinical photograph and the substantive description of the depicted condition is not itself evidence of educational inadequacy; cross-referencing may be an appropriate component of educational design [11,12,32-35]. The observation establishes only the documented relationship among the photograph, the identified condition, the textbook structure, the location of the substantive clinical description, and the accompanying reference.

The reconstruction was subsequently extended beyond the 2005-2019 publication sequence to distinguish later events in the educational life cycle of the material: discovery and formal objection by the person depicted, contemporary inquiry into documentation supporting continued use, institutional reassessment, modification of access to existing educational material, and continued curricular use of existing editions in 2026. These events are analytically distinct from publication or reproduction because they concern material already in circulation.

Following discovery of continued circulation in 2023, the person depicted raised formal objections to further use during 2023-2025. The person depicted, who is also the author of this viewpoint, reports that she did not provide consent for the educational publication or repeated reuse of these childhood photographs. Separately, in 2025, an institutional inquiry established that the publisher could not produce the requested consent documentation concerning the photographed persons and described reliance on contractual declarations made by authors. These are analytically distinct observations: the first concerns the reported absence of authorization by the person depicted; the second concerns the inability of the publisher, during contemporary inquiry, to produce documentation establishing the basis and scope of authorization for continued use. Both constitute information relevant to contemporary reassessment.

Institutional responses were not uniform. At Pomeranian Medical University in Szczecin, identified editions and reprints were withdrawn from the university library collection and catalogue, with 146 copies withdrawn in total, and relevant recommended-reading lists were subsequently revised. At the same time, existing editions from the analyzed textbook lineage remained formally incorporated into medical and health-professions curricula in other educational settings in 2026.

These later events distinguish publication persistence from curricular persistence and reassessment. Continued designation of an existing textbook as basic, mandatory, recommended, or supplementary literature does not constitute a new publication or reproduction; it demonstrates that previously published material may remain part of contemporary education after the publication sequence has ended. The longitudinal sequence and subsequent reassessment events are summarized in Table 1.

A complete item-level reconstruction of the 28 documented editions and reprints from 2005 to 2019, together with the subsequent objection, authorization inquiry, institutional response, and curricular persistence, is provided in Tables S1 and S2 in Multimedia Appendix 1.

This longitudinal reconstruction provides the observational basis for the concepts of educational disconnection and educational value decay developed in the sections “The Problem of Educational Disconnection” and “Educational Value Decay and the Persistence of Clinical Images”; it does not itself establish absence or loss of educational value. Material changes in the information available to those responsible for continued use may provide a reason for reassessment without predetermining its outcome.

The observation is problem-generating and concept-generating rather than representative. It establishes the longitudinal basis for the question examined in this viewpoint but does not establish the prevalence of educational disconnection beyond the analyzed material.

**Table 1:** Persistence of the image-condition configuration across 28 documented editions and reprints and subsequent reassessment events, 2005-2026.

Year/period	Documented event	Status of the clinical image or material	Analytical significance
2005	First edition of the analyzed textbook lineage	Highly sensitive pediatric clinical photograph included	The depicted clinical condition was identified beside the photograph. The condition was not identified in the table of contents as part of the chapter containing the photograph; the accompanying text stated that the condition was described elsewhere, and its substantive clinical description was located at that other curricular location.
2005-2006	Subsequent reprints of the first edition	Same photograph reproduced	The same documented image-condition configuration was retained.
2006-2010	Subsequent documented editions and reprints	Same photograph retained	The observable relationship among image location, condition identification, declared curricular location, substantive clinical description, and the destination indicated in the accompanying text persisted.
2015	Second edition	Same photograph retained in a new edition	The same documented image-condition configuration was retained in the new edition.
2015-2019	Subsequent reprints of the second edition	Same photograph reproduced	The same documented image-condition configuration persisted across subsequent reprints.
2019	Last identified reprint within the analyzed 28-item textbook sequence	Same photograph remained present	The documented image-condition configuration remained present at the end of the 2005-2019 longitudinal sequence.
2023-2025	Discovery of continued circulation followed by formal objection by the person depicted	The person depicted became aware of continued use and objected to further use of the photograph	New information entered the educational life cycle of the image, creating a documented external trigger for reassessment; this was not a new publication event.
2025	Institutional inquiry into documentation supporting continued use	The publisher stated that it did not possess the requested consents concerning the photographed persons and described reliance on contractual declarations made by authors	The publisher was unable to produce the requested consent documentation during contemporary inquiry. This constituted new information relevant to reassessment of the conditions supporting continued use.
2025	Institutional reassessment and withdrawal at Pomeranian Medical University in Szczecin	Identified editions and reprints were withdrawn from the university library collection and catalogue; 146 copies were withdrawn in total	Documented example of an external reassessment trigger followed by institutional review and modification of access to existing educational material.
2026	Review and amendment of teaching syllabi at Pomeranian Medical University in Szczecin	Identified textbooks were removed from relevant recommended-reading lists	Documented example of reassessment leading to modification of formal curricular recommendations.
2026	Continued curricular use of existing editions in other educational settings	Existing editions remained designated as basic, mandatory, recommended, or supplementary literature according to the respective institution	Previously published material remained incorporated into contemporary medical and health-professions education after the objection; this represents curricular persistence rather than a new publication or reproduction event.

### Author Positionality

The author holds a doctorate in medical and health sciences and is a psychologist, psychotherapist, psychotraumatologist, educator, clinician, and researcher whose work includes patient rights, trauma, medical ethics, and health care governance. At the same time, she is the person depicted in the pediatric clinical photographs whose long-term educational use provided the starting point for the questions developed in this viewpoint. This dual position—as both a researcher and the person depicted in the analyzed photographs—made it possible to identify not only the continued presence of highly sensitive images in educational material, but also their relationship to the curricular structure and substantive clinical teaching. This perspective contributed to the formulation of this paper’s central question: “What additional educational function does a highly sensitive image serve at the point of its presentation when the textbook’s declared curricular structure, substantive teaching of the depicted condition, and accompanying cross-reference direct the reader elsewhere?”

The analysis does not seek to determine intentions or evaluate the decisions of specific authors, editors, publishers, or institutions. It is limited to observable relationships within the educational material, the scope of patient information disclosed, and the identifiable educational function of the image.

### The Problem of Educational Disconnection

 Educational disconnection  refers to a situation in which a highly sensitive clinical image remains present in educational material, but its  image-specific educational contribution at the point of presentation is difficult to reconstruct from the observable relationship among the image, the clinical condition identified with it, the condition’s declared curricular location, the location of its substantive clinical description, and the destination indicated in the text accompanying the photograph .

Physical separation of an image from the substantive clinical description of the depicted condition does not in itself constitute educational disconnection.  Cross-referencing  between chapters may be an appropriate component of educational design, and a photograph may serve a distinct educational function outside the location in which the depicted condition is substantively described  [11,12,32-35] . The defining issue is therefore not simply the physical “distance” between an image and related clinical content. It arises when  the condition is identified beside the photograph, while the accompanying text states that the condition is described elsewhere and its substantive clinical description is located at that other curricular location, yet the image-specific educational contribution at the point of presentation remains difficult to identify .

The distinction is important because  identification of the depicted condition, identification of where that condition is substantively described, and identification of the educational contribution of its photograph are analytically distinct . A photograph may identify what condition is depicted; the textbook’s declared curricular structure may indicate where that condition is addressed; and the accompanying text may explicitly state that the condition is described elsewhere. None of these elements alone establishes  what additional educational information the photograph itself contributes at the location in which it is presented .

The relevant analytical question is therefore not merely whether the photograph depicts a clinically identifiable condition, but “What additional educational information does this particular photograph contribute here?” Educational disconnection does not mean that the photograph lacks educational value, that its location is inappropriate, or that continued use is unjustified. Nor does it permit reconstruction of the intentions, learning objectives, or educational reasoning of the author, editor, or publisher. It is an  epistemic and decision-making signal  indicating that the image’s context-specific educational contribution requires clarification.

If that contribution can be identified—for example, recognition of a phenotype, distribution of clinical features, body proportions, diagnostic differentiation, or an anatomical relationship relevant to the educational context—the photograph may remain well justified even when the substantive clinical description of the depicted condition is located elsewhere  [1,3,7-10] . In such a case, the apparent disconnection may be resolved by identifying the distinct educational contribution made by the image at its actual point of presentation.

If, however,  the image-specific educational contribution at that location cannot be identified , reassessment of the image’s function, location, or continued use is warranted. Reassessment does not predetermine removal. Its purpose is to determine whether a context-specific educational justification can be established.

Only after the image’s educational contribution has been identified can  exposure - function proportionality  be meaningfully assessed. Nudity, intimate anatomy, or full-body presentation may be educationally necessary when these features convey information required for recognition of phenotype, anatomical relationships, distribution of clinical findings, or another specified educational objective  [1,3,7-10] . Conversely, where additional exposure contributes no information necessary to the identified function, its inclusion requires separate justification.  The relevant standard is therefore not minimum exposure in the abstract, but exposure proportional to the educational information that the image contributes at the point of presentation. 

Educational disconnection is therefore neither a stand-alone educational theory nor an empirically validated measurement category. It is a  conceptual tool introduced in this viewpoint for identifying situations in which the relationship among  the clinical photograph, the condition identified with it, the condition’s declared curricular location, the location of its substantive clinical description, the destination indicated in the accompanying text, the image-specific educational contribution, and the scope of patient exposure  requires clarification and, where necessary, reassessment.

### Educational Value Decay and the Persistence of Clinical Images

Educational disconnection is primarily contextual: it concerns whether  the educational contribution of an image can be identified at the point of presentation in relation to the observable image - condition configuration . Longitudinal persistence introduces a different question: “ If the same photograph remains within the same image–condition configuration across successive editions and reprints, does the context-specific justification for its presence at that particular location remain current over time?” 

Neither the passage of time nor repeated reproduction demonstrates loss of educational value. A photograph depicting a characteristic phenotype, anatomical feature, or distribution of clinical findings may remain educationally valuable for many years [1-3,23]. The persistence of the image is therefore not itself the problem; the relevant question concerns the persistence of its context-specific educational justification. 

Educational value depends on the relationship among  what the image shows, what educational information it provides, where and how it is presented, and the educational objective it serves [11-13,31-35,41]. The photograph itself may remain unchanged while its educational context changes through revised objectives, relocation or redistribution, alternative representations, or a different technological or distribution environment.

In this viewpoint, educational value decay  refers to the possibility that the relationship between a clinical image and the context-specific educational justification for its presence at a particular location may weaken over time, even while the image itself continues to be reproduced or used. 

The term does  not  mean that an older photograph is less educationally valuable or that repeated reproduction progressively diminishes its value. It describes a possible divergence between  persistence of the image and persistence of the justification for its particular educational use . These trajectories may remain fully aligned, in which case continued use may remain well justified.

Educational value decay becomes a question for reassessment when a material change affects the image’s educational function, location, available alternatives, or conditions of continued use,  including information relevant to the basis or scope of authorization for that use .  The concept therefore identifies a possibility requiring evaluation, not a conclusion that educational value has actually declined. 

The distinction between the two constructs can be expressed through two questions: (1) educational disconnection: “What additional educational information does this image contribute here, when the condition identified with it is substantively described elsewhere in the material?” (2) educational value decay: “Does the context-specific justification for the presence of this image here remain current at the point of subsequent use?” 

The first question is primarily  contextual ; the second is  temporal .

For highly sensitive pediatric photographs, this temporal distinction has particular significance because the information disclosed by the image remains embedded in the photograph while the educational and technological context surrounding its use may change [1,7,8,15,17-20,42] . Reassessment therefore asks  whether the scope of exposure continues to correspond to the image-specific educational contribution at the point of current use , rather than assuming that sensitivity, age, or repetition makes continued use inappropriate.

Accordingly, educational value decay is not an empirically validated measurement category or evidence that educational value necessarily diminishes over time. It is a  conceptual tool introduced in this viewpoint for identifying when longitudinal persistence creates a reason to reassess whether an image’s context-specific educational justification remains current.

### A Practical Educational Reassessment Framework for Highly Sensitive Pediatric Clinical Images

The  Educational Reassessment Framework translates the preceding concepts into a practical, sequential decision-making process. It begins not with the age, number of reproductions, or sensitivity of the photograph, but by asking  what educational contribution the image provides at the specific point at which it appears . The framework proceeds from identifying what the photograph depicts and what educational information it is expected to provide, through assessment of its relationship to the observable image - condition configuration and its image-specific educational contribution  at that location, to  exposure - function proportionality , equivalent alternatives, continuing validity of justification, conditions of responsible use, and a documented decision.

The framework is neither intended to classify photographs automatically as educational or noneducational, nor does it presume that historical, repeatedly used, or highly sensitive images should be withdrawn. Its purpose is to make the reasoning supporting continued use  identifiable, current, context-specific, and reconstructable .

### When Should Reassessment Be Triggered?

 Educational disconnection is one trigger for reassessment.  It arises when the photograph and the clinical condition identified with it can be observed, but the image-specific educational contribution at the point of presentation remains difficult to reconstruct from the relationship among  image location, condition identification, the condition’s declared curricular location, the location of its substantive clinical description, and the destination indicated in the accompanying text .

Reassessment may also be triggered by a  material change in the educational life cycle of the image , such as a new edition, substantial content revision, relocation of the image or change in its educational context, change in audience or mode of distribution, migration to a digital environment, emergence of an equally effective but less intrusive alternative, or inability to reconstruct the previous justification for use [1-3,15-20,23,42-44] .

New material information may likewise provide a trigger, including a documented objection or request to discontinue use by the person depicted, newly identified information concerning the conditions under which the image was obtained or authorized, or inability during contemporary review to reconstruct or produce relevant authorization documentation  [1,7-10,15,19-24,45] .

These events do not predetermine the outcome of reassessment.  They alter the information or context relevant to the decision and therefore create a reason to reconsider whether the existing justification remains applicable.  Not every technical reproduction requires repetition of the entire process. Reassessment is relevant when a decision concerning continued use is made or when a material change affects the context in which the image is expected to serve its educational function.

### Who Should Perform the Reassessment?

Responsibility should be located as close as practicable to the decision concerning use of the image. Depending on the educational setting, this may be the author, content editor, educational team, publisher, or another entity responsible for approving or continuing its use.

Multiple parallel assessments are unnecessary. What is required is an  identifiable point of responsibility  capable of linking the image’s educational function, its location within the educational material, the scope of patient information disclosed, the conditions of use, and the resulting decision.

The framework comprises  8 sequential stages  (Table 2). The first 3 stages establish  what condition the image depicts, where that condition is located and substantively described within the material, and what additional educational information the image contributes at its actual point of presentation . Only after these questions have been addressed can exposure-function proportionality, equivalent alternatives, continuing validity of justification, and conditions of responsible use be meaningfully assessed.

**Table 2:** Educational reassessment framework: from clinical identification to context-specific educational justification.

Assessment stage	Decision question	Assessment criterion/possible action	Responsible entity
1. Educational function	What educational information is this image expected to provide?	Identify the specific information or educational function provided by the image itself, such as recognition of a phenotype, distribution of clinical findings, an anatomical relationship, diagnostic differentiation, or treatment outcome. Identification of the depicted clinical condition alone does not establish the educational function of the image [5,11-14,32-35,41,46].	Author or content editor
2. Image-condition configuration	Where does the material locate and substantively describe the condition identified with the photograph, and how does the image location relate to those locations?	Examine image location, condition identification, declared curricular location, location of substantive clinical description, and the destination indicated in the accompanying text. Separation among these elements is not inherently problematic; the purpose of this stage is to establish the observable configuration before inferring an educational function.	Author, content editor, or educational team
3. Contribution at the point of presentation	What additional educational information does this particular image contribute here?	Identify the image-specific educational contribution at the point of presentation. Where the accompanying text states that the depicted condition is described elsewhere, determine what educational information the photograph contributes at its present location beyond identification of the condition. If a context-specific contribution can be identified, the image location may remain justified. If it remains difficult to identify, *educational disconnection *signals the need for reassessment of function, location, or continued use.	Author, content editor, or educational team
4. Exposure-function proportionality	Is the scope of patient information disclosed necessary to fulfill the function identified in stages 1-3?	Retain full-body context, intimate anatomy, or other highly sensitive elements when they are necessary to convey the specified educational information. Where part of the exposure contributes no information necessary to the identified objective, consider limiting it without removing clinically relevant educational information [1,3,7-10,15].	Author or content editor
5. Equivalent alternative	Can the same context-specific information be conveyed equally effectively through a less intrusive representation?	Compare alternatives against the same identified educational function, rather than solely against degree of exposure. If a less intrusive representation preserves equivalent educational information, it should be considered. If it does not, continued use of the existing image may remain educationally justified, subject to the separate assessment of the conditions of responsible use in stage 7 [1,3,4,11-15,31,46].	Author, editor, or educational team
6. Temporal validity of justification	Does the context-specific justification for the presence of this image here remain current at the next material point of use?	Consider whether changes in image function, material structure, location, educational objectives, audience, available alternatives, or distribution environment affect the previous justification. Prior publication alone does not establish that the same context-specific justification remains current. This stage operationalizes the temporal question associated with *educational value decay*.	Entity responsible for continued use
7. Conditions of responsible use	Are the current conditions of image use, including the basis and scope of authorization, appropriate for the intended use?	Independently assess the basis for use, consent or assent where applicable, the documented scope of authorization in relation to the current use, documented objection or withdrawal request where relevant, privacy, identifiability, the availability and reconstructability of relevant authorization documentation, and the distribution environment [1,7-10,15-24,42-45,47-49]. Prior publication or repeated use alone should not be treated as evidence that the current use falls within the scope of an earlier authorization. Educational value does not substitute for appropriate conditions of use, and an appropriate basis for use does not itself establish educational necessity at a particular location.	Publisher or other entity responsible for use
8. Documented decision	Can it later be reconstructed why the image was retained, modified, relocated, replaced, or withdrawn?	Maintain a concise record of the decision sequence: educational function→image-condition configuration→contribution here→exposure-function proportionality→alternatives→temporal validity→conditions of use→decision.	Entity approving use

The framework should be understood as  a sequence rather than a collection of independent criteria . In particular, neither can exposure-function proportionality nor the equivalence of an alternative be meaningfully assessed until it has first been established  what condition the image depicts, where that condition is located and substantively described within the material, and what additional educational information the photograph contributes at its point of presentation .

This ordering is important. Asking whether a photograph reveals “too much” before establishing what information it needs to convey reverses the analytical sequence. Similarly, asking whether a less intrusive alternative is equivalent requires first identifying  the educational function against which equivalence is to be assessed .

A discrepancy between image location and the location in which the depicted condition is substantively described does not predetermine the outcome of reassessment. An image positioned elsewhere may serve a distinct and well-justified educational function—for example, comparison, phenotype recognition, diagnostic differentiation, or illustration of a cross-cutting clinical feature  [11,12,32,34,35,41,46] . The framework requires only that  the function be identifiable and linked to the point at which the image is presented .

The outcome of reassessment is therefore not predetermined. If a photograph serves an identifiable educational function at the point of presentation, is meaningfully integrated into the educational material, and requires its full scope of exposure to convey that information,  and if the conditions of responsible use—including the basis and scope of authorization—remain appropriate , reassessment may  confirm continued use of the image without modification . In other circumstances, reassessment may support improved integration with the surrounding content, modification of location or caption, reduction of exposure, use of an equally effective alternative, or discontinuation of further use.

The framework therefore neither asks whether a highly sensitive photograph should remain  because it has been used before nor whether it should be removed  because it is old, repeatedly reproduced, or highly sensitive . It asks whether its continued use can be supported by a  current, identifiable, and context-specific educational justification, together with appropriate conditions of responsible use, including an appropriate basis and scope of authorization for the intended use .

## Discussion

### Overview

The analysis suggests that evaluation of highly sensitive pediatric clinical photographs should begin with  the relationship between the observable image - condition configuration and the educational contribution of the photograph at its point of presentation . Identification of the depicted clinical condition, identification of where that condition is substantively described, and identification of the educational contribution of its photograph are analytically distinct. The relevant question is  what additional educational information the photograph itself contributes at the point of its actual presentation .

This is the function of  educational disconnection  as a conceptual signal. It does not imply that a photograph is unnecessary, inappropriate, or devoid of educational value. Rather, reassessment serves to  clarify the image-specific educational contribution , not to confirm a predetermined assumption of inappropriate use.

### Proportionality Follows Identification of Educational Function

The scope of exposure cannot be meaningfully assessed until the image’s educational function has been identified. Nudity, intimate anatomy, full-body presentation, or other highly sensitive bodily features may be necessary to convey clinically relevant information  [1,3,7-10] . Minimization should therefore not mean simply “show as little as possible,” but  disclosing the scope of patient information necessary to preserve the information required by the identified educational function .

The resulting principle is that the scope of exposure can be assessed as proportionate only in relation to an identified educational contribution. 

If full exposure provides information necessary to achieve the identified educational objective, retaining it may be justified  [1,3,7-10] . Conversely, if additional exposure contributes no information necessary to that function, its inclusion requires separate justification. The relevant comparison is between  the patient information disclosed and the educational information contributed by that disclosure .

### Reuse Is Not a Problem in Itself

 Reuse  is not an indicator of inappropriate use or loss of educational value. A high-quality photograph, particularly one depicting a rare or difficult-to-document condition, may retain substantial educational value for many years, and responsible reuse may reduce the need to photograph additional patients  [1-3,23] .

This potential ethical advantage of reuse, however, depends on the conditions under which the existing image continues to be used. Avoiding unnecessary photography of additional patients should not be achieved by concentrating the cumulative burden of repeated exposure on a single individual who did not authorize that use. Responsible reuse should therefore protect both patients who might otherwise be newly photographed and the person whose existing image is repeatedly reproduced.

This distinction becomes particularly important when the same image persists across successive editions, formats, distribution environments, or educational settings over extended periods. In such circumstances, an individual may effectively become an enduring visual representation of a clinical condition. Where that representational role was not authorized by the person depicted, the fact that continued reuse may reduce the need to photograph additional patients does not, by itself, provide an ethical justification for continued exposure.

The significance of longitudinality is therefore not the number of reproductions itself. Educational value decay asks whether  the context-specific justification for the image’s presence remains current . Repeated use becomes relevant to reassessment when a  material trigger  changes the educational context, the conditions of responsible use, or the information available to the entity responsible for continued use. The longitudinal question is therefore not  “Has this image been used too many times?”  but  “Does the justification for this particular use remain current?” 

### Consent and Educational Function Are Distinct Dimensions of Assessment

Consent, assent, privacy, and protection against identification remain fundamental conditions of responsible clinical-image use  [1,7-10,15,19-22,24,45,47-50] , but they answer a different question from educational function.

An image may have substantial educational value while the conditions supporting a particular use require reassessment. Conversely, an appropriate basis for use does not establish why a particular photograph is educationally necessary at a particular location.  Educational justification and authorization should therefore be evaluated as related but analytically distinct dimensions. 

Historical consent, where documented, should not automatically be treated as establishing authorization for every subsequent form of use.  The existence of consent, its documented scope, and the specific later use are distinct questions.  Previous lifecycle-governance analysis has identified the importance of durable consent documentation and verification checkpoints before republication, digitization, or educational redistribution  [40] . Changes in educational context, audience, distribution environment, or mode of dissemination may therefore require consideration of whether a subsequent use remains appropriately supported  [1,7-10,15,19-22,24,45] .

For pediatric photographs, this assessment may additionally require consideration of assent where developmentally applicable and later preferences or objections expressed by the person depicted.  Prior publication or repeated use alone should not be treated as evidence that every later reproduction, redistribution, or educational use falls within the scope of an earlier authorization. 

The potential educational or ethical advantage of reusing an existing image—including avoidance of unnecessary photography of additional patients—does not substitute for establishing that the continued use of that particular image remains appropriately authorized. Educational value, rarity, image quality, or previous use cannot by themselves answer the separate question of whether the person depicted authorized the relevant use.

Two questions should therefore remain distinct: “Can we use this image?” and “Why do we need this particular image, here, and with this scope of exposure?” 

The first concerns  conditions of responsible use ; the second concerns  educational function, image-specific contribution, and exposure - function proportionality . The Educational Reassessment Framework does not replace requirements concerning consent, assent, privacy, authorization, or patient protection; it adds a distinct level of  educational justification .

In digital environments, protection against identification must also extend beyond the face. Distinctive bodily features, scars, birthmarks, rare phenotypes, or contextual information may enable recognition, while copying, searching, screenshotting, and secondary dissemination may alter the scale and persistence of exposure  [1,15-18,42-44] . Digital redistribution may therefore change relevant conditions of use even when the underlying photograph remains unchanged.

### The Significance of Educational Architecture

The proposed analysis extends beyond the conventional image-text relationship to  the relationship between the photograph, the clinical condition identified with it, and the observable structure of the educational material . Educational design literature supports attention to relationships among instructional content, learning objectives, organization, and the integration of educational elements  [11-13,31-35,51] .

This does not require linear educational materials or placement of every photograph alongside the substantive clinical description of the depicted condition. An image may be educationally valuable precisely because it appears elsewhere—for example, as a comparison, an example of phenotype, an element of differential diagnosis, or an illustration of a cross-cutting topic  [11,12,32,34,35,41,46] .  Such a context-specific function should, however, be identifiable. 

The issue is therefore not  “an image appears in another chapter,”  but whether  its presence at that location provides an identifiable additional educational contribution . An accompanying reference may indicate where the depicted condition is substantively described without establishing what the photograph contributes at its actual location.

### Scope of the Proposed Argument

This viewpoint supports a  conceptual rather than empirical generalization . Its contribution is to distinguish four questions: (1) “What clinical condition is identified with the image?” (2) “Where is that condition substantively described within the educational material?” (3) “What additional educational information does the image contribute at the point of its actual presentation?” and (4) “Is the scope of patient information disclosed necessary for that specific contribution?” 

These questions correspond respectively to  clinical identification, image - condition configuration, image-specific educational contribution, and exposure - function proportionality .

The broader implication is that  continued presence, continued educational justification, and authorization for continued use are related but distinct questions . A photograph may remain educationally appropriate in its original form and location when its current educational contribution can be identified, and the scope of information disclosed is proportionate to that contribution;  continued use additionally requires that the applicable conditions of responsible use, including the basis and scope of authorization, remain appropriately supported. 

### Limitations

This viewpoint has several limitations that define the scope of the proposed argument.

First, its starting point is a  documented longitudinal observation of a single academic textbook lineage , rather than a representative sample of medical textbooks or a systematic review of educational resources. The persistence of the analyzed  image - condition configuration  across  28 documented editions and reprints between 2005 and 2019 provides a longitudinal basis for formulating a conceptual problem but does not allow estimation of the prevalence of educational disconnection in medical education.  The present argument therefore supports conceptual rather than empirical generalization. 

Second, the analysis concerns the  observable architecture of educational material , rather than direct learning outcomes. Neither were experiments involving learners conducted, nor were learning outcomes compared with and without the analyzed photographs. The analysis therefore cannot establish whether a photograph possessed a particular degree of educational value; it can establish only the observable relationship between the image, the condition identified with it, its location within the material, and its image-specific educational contribution.

Third, the complete process of educational design and use cannot be reconstructed from the final educational product. Authors, editors, or publishers may have had educational objectives or reasons for image placement that are not visible to the reader, while learners may use chapters in different sequences, consult other sections, or receive additional context from educators  [11,12,31-35] . The analysis is therefore limited to the  observable image - condition configuration and does not infer undocumented intentions, learning objectives, or educational reasoning.

Fourth,  educational disconnection  is a conceptual construct rather than an empirically validated measurement category. Further operationalization is required to determine whether independent researchers or educators can reliably identify and assess it. The construct should therefore be understood as a conceptual and decision-making signal rather than a diagnostic classification of educational material.

Fifth,  educational value decay  is a temporal extension of the proposed analysis rather than a claim that educational value declines over time. Persistence of the analyzed  image - condition configuration  across the longitudinal sequence cannot itself establish that an earlier educational justification weakened or became outdated. Determining whether that justification remains current requires assessment at subsequent points of use.  Persistence of educational justification does not establish that the conditions authorizing continued use remain appropriate; educational justification and authorization are assessed as distinct dimensions in the proposed framework. 

Sixth, the  Educational Reassessment Framework  is a proposed normative and practical decision-making tool rather than a validated measurement instrument. Its sequential logic requires further evaluation for feasibility, interuser agreement, reproducibility, and usefulness across specialties and educational settings.

Seventh, the analysis focuses on  highly sensitive pediatric clinical photographs , for which the relationship between educational function and scope of patient exposure has particular significance. Its broader applicability to other illustrations used in medical education requires further study.

Finally, the author occupies a dual position as both researcher and the person depicted in the photographs that initiated the analysis. This position contributed to identifying the relevant relationships within the educational material but may also influence conceptualization of the problem. The analysis therefore distinguishes  observable properties of the educational material  from inferences concerning intentions, motives, learning objectives, or undocumented decision-making processes. The proposed constructs and framework should be examined by independent researchers and educators using materials beyond the case from which they were developed.

These limitations define a direct research agenda. A next step should be a  systematic empirical study of a larger corpus of educational materials , independently coding  image location, condition identification, declared curricular location, substantive clinical description, cross-reference destination, image-specific educational contribution, and scope of patient exposure . Such research could estimate the prevalence and forms of educational disconnection, test its operationalizability and interrater reliability, examine whether educational value decay can be operationalized longitudinally, and evaluate the feasibility and reproducibility of the  Educational Reassessment Framework  across educational settings.

### Conclusions

Clinical photography can be an indispensable tool in medical education, including when it depicts nudity, intimate anatomy, or other highly sensitive bodily features of a child. Neither a high degree of exposure nor repeated use of an image determines, in itself, its educational value or the justification for its continued use.

The central distinction proposed in this viewpoint is that  identification of the depicted clinical condition, identification of where that condition is substantively described, and identification of the educational contribution of the photograph are distinct elements . A separate question therefore remains: “ What additional educational information does this photograph contribute at the point of its actual presentation?” 

Educational disconnection identifies situations in which this image-specific contribution is difficult to reconstruct from the observable  image - condition configuration . It does not imply that the photograph lacks educational value or that its use or location is inappropriate; rather, it signals the need to clarify  what the image contributes here and why it is needed at this particular location .

When the same  image - condition configuration  persists across successive editions and reprints, educational value decay adds a temporal question:  “Does the context-specific justification for the image’s presence remain current?”  The concept identifies the possibility that  persistence of the photograph and persistence of its context-specific educational justification may cease to remain aligned , without assuming that educational value necessarily declines over time or through repeated reproduction.

The  Educational Reassessment Framework  translates these questions into a practical decision-making process: “ What clinical condition is identified with the image?”→“Where is that condition substantively described within the material?”→“What additional educational information does the image contribute here?”→“Why is this particular image needed at this particular location?”  Only then can the proportionality of patient exposure, equivalent alternatives, continuing validity of justification, and  conditions of responsible use, including the basis and scope of authorization , be meaningfully assessed.

Reassessment is not a mechanism for the automatic removal of historical, repeatedly used, or highly sensitive clinical photographs.  Responsible reuse of a high-quality image may remain educationally valuable and may reduce the need to photograph additional patients. However, educational value and the potential benefits of reuse do not substitute for appropriate authorization of the continued use of the existing image.  Reassessment may therefore confirm continued use, including the full scope of exposure, when supported by both an  identifiable, current, and context-specific educational contribution and appropriate conditions of responsible use .

The final question is therefore not simply, “Can this image remain?” It is “What additional educational information does this image contribute here, why is this particular image needed at this particular location, is the scope of patient information it discloses necessary and proportionate to that specific educational contribution, and are the conditions supporting its continued use appropriately established?”

## Appendix

Multimedia Appendix 1 Detailed longitudinal reconstruction of 28 editions and reprints of a single academic textbook lineage and subsequent reassessment and curricular persistence, 2005-2026.
