# Supplementary material for: Is This Still Medical Education? The Educational Use of Nude Pediatric Clinical Photographs: Bioethical Reassessment
Source: JMIR Med Educ. 2026 Sep 16;12:e104023. doi: 10.2196/104023 (PMC13628035; doi:10.2196/104023)
Supplement: Supplementary file 1 [file mededu_v12i1e104023_app1.docx]

### Scope and Purpose of the Reconstruction

The broader reconstruction underlying this Viewpoint identified the same pediatric clinical photographs across **more than 40 publications and educational or digital resources**, including books and textbook publications, conference materials, e-books, PDFs, presentations, and other educational or digital formats. This wider dissemination trajectory provides the broader context for the present analysis and has been reported previously **[1]**.

For the purposes of the present Viewpoint, the longitudinal analysis was narrowed to a **single academic textbook lineage**. The textbook title, chapter numbers, page numbers, names of the clinical conditions depicted, and other identifiers that could unnecessarily facilitate retrieval of the underlying highly sensitive pediatric photographs are not reproduced in this public Appendix. Full bibliographic and documentary information supporting the reconstruction can be provided for editorial verification.

This textbook lineage was selected because it provided a bounded and relatively stable context in which the relationship among **the clinical photographs, the conditions identified with them, the declared curricular locations of those conditions, the locations of their substantive clinical descriptions, and the destinations indicated in the text accompanying the photographs** could be followed longitudinally.

Within this textbook lineage, **28 documented editions and reprints containing the analyzed highly sensitive pediatric clinical photograph were identified between 2005 and 2019**. The detailed reconstruction in **Table S1** follows these 28 items individually.

The distinction between the broader trajectory and the analyzed textbook lineage is important. The **more than 40 publications and educational or digital resources** document the wider dissemination of the pediatric clinical photographs, whereas the **28 editions and reprints of the single textbook lineage** constitute the bounded longitudinal sequence used in this Viewpoint to examine persistence of a specific **image–condition configuration**. The number 28 therefore refers specifically to documented editions and reprints within this textbook lineage and should not be interpreted as the total number of publications or resources in which the photographs were identified.

Across these 28 documented editions and reprints, the analyzed photograph remained present within the same observable **image–condition configuration**. At the point of image presentation, the depicted clinical condition was **identified by name, but no substantive clinical description of that condition was provided there**. The condition was not identified in the textbook’s table of contents as a component of the chapter containing the photograph. Instead, the text accompanying the photograph explicitly stated that the condition was described elsewhere in the textbook. Its substantive clinical description was located at that other curricular location.

For the purposes of this reconstruction, **image–condition configuration** refers descriptively to the observable relationship among:

1. the **clinical photograph**;
2. the **clinical condition identified by name at the point of image presentation**;
3. the condition’s **declared curricular location** within the textbook structure;
4. the location of its **substantive clinical description**; and
5. the **destination indicated in the text accompanying the photograph**.

The term is descriptive rather than interpretive. It does not attribute an educational intention, learning objective, or rationale for image placement to the authors, editors, or publisher. Nor does persistence of this configuration establish that the photograph lacked educational value, that its placement was inappropriate, or that repeated use was itself problematic.

The longitudinal reconstruction examines **the persistence of this observable relationship across the 28-item textbook sequence**. Repeated reproduction itself is not treated as evidence of diminished educational value, inappropriate placement, or inappropriate use.

### Identification and Verification of Editions and Reprints

The 28 editions and reprints were identified and cross-checked using **multiple documentary sources**, depending on the availability of individual items. These included:

- **direct examination of physical copies and publication imprints**;
- **institutional library holdings and catalogue records**;
- **online bibliographic records associated with second-hand and antiquarian booksellers**; and
- **online marketplace records containing identifiable bibliographic information concerning particular editions or reprints**.

Individual editions and reprints were therefore **not necessarily identified or verified through the same source type**. Where available, physical copies and institutional library documentation provided direct verification. For other items, bibliographic reconstruction was based on identifiable publication information available through online book-trade, antiquarian, marketplace, or other bibliographic records.

The **Evidence** column in **Table S1** records the source category through which each item was identified or verified. These categories should not be interpreted as indicating that every edition or reprint was physically examined. Rather, they provide a transparent record of the evidentiary basis used to reconstruct the longitudinal textbook sequence.

For consistency, the following evidence categories are used:

- **Physical copy / publication imprint** — the relevant edition or reprint was directly examined and its publication information verified from the physical item;
- **Institutional library record** — the edition or reprint was identified or verified through institutional library holdings, catalogue records, or other library documentation;
- **Online antiquarian / second-hand bibliographic record** — the edition or reprint was identified through an online antiquarian or second-hand bookseller record containing relevant bibliographic information;
- **Online marketplace bibliographic record** — the edition or reprint was identified through an online marketplace record containing relevant bibliographic information; and
- **Bibliographic reconstruction from multiple sources** — the edition or reprint was reconstructed by comparing bibliographic information across more than one documentary source.

Where more than one independent source was available for the same item, the relevant evidence categories are reported together.

### Temporal Scope Beyond the 28 Editions and Reprints

The reconstruction was subsequently extended beyond the 2005–2019 publication sequence to examine later events in the educational life cycle of the material: **discovery and formal objection by the person depicted; contemporary inquiry into documentation supporting continued use; the publisher’s response concerning the requested consent documentation; institutional reassessment and withdrawal; revision of curricular recommendations; and continued curricular use of existing editions in other educational settings**.

These later events are analytically distinct from the **28 editions and reprints**. They concern material already in circulation and are therefore **not counted as additional publication or reproduction events**.

They are presented separately in **Table S2** to distinguish:

**publication persistence → subsequent reassessment events → curricular persistence.**

## **Table S1.** Detailed longitudinal reconstruction of 28 documented editions and reprints, 2005-2019.

| No. | Year | Documented event | Status of image or material | Observed image–condition configuration / analytical significance | Evidence |
| --- | --- | --- | --- | --- | --- |
| **1** | 2005 | First edition | Highly sensitive pediatric clinical photograph included | The clinical condition was identified by name at the point of image presentation, but no substantive clinical description of that condition was provided there. The condition was not identified in the table of contents as a component of the chapter containing the photograph. The accompanying text stated that the condition was described elsewhere in the textbook, and its substantive clinical description was located at that other curricular location. | Bibliographic reconstruction; institutional library record |
| **2** | 2005–2006 | First-edition reprint I | Same photograph reproduced | Same documented **image–condition configuration** retained | Bibliographic reconstruction |
| **3** | 2005–2006 | First-edition reprint II | Same photograph reproduced | Same documented **image–condition configuration** retained | Bibliographic reconstruction |
| **4** | 2005–2006 | First-edition reprint III | Same photograph reproduced | Same documented **image–condition configuration** retained | Bibliographic reconstruction |
| **5** | 2005–2006 | First-edition reprint IV | Same photograph reproduced | Same documented **image–condition configuration** retained | Bibliographic reconstruction |
| **6** | 2005–2006 | First-edition reprint V | Same photograph reproduced | Same documented **image–condition configuration** retained | Bibliographic reconstruction |
| **7** | 2005–2006 | First-edition reprint VI | Same photograph reproduced | Same documented **image–condition configuration** retained | Bibliographic reconstruction |
| **8** | 2005–2006 | First-edition reprint VII | Same photograph reproduced | Same documented **image–condition configuration** retained | Bibliographic reconstruction |
| **9** | 2005–2006 | First-edition reprint VIII | Same photograph reproduced | Same documented **image–condition configuration** retained | Bibliographic reconstruction |
| **10** | 2005–2006 | First-edition reprint IX | Same photograph reproduced | Same documented **image–condition configuration** retained | Bibliographic reconstruction |
| **11** | 2006 | Subsequent documented reprint | Same photograph retained | Continued use within the same documented **image–condition configuration** | Institutional library record |
| **12** | 2007 | Subsequent documented reprint | Same photograph retained | Continued use within the same documented **image–condition configuration** | Bibliographic reconstruction |
| **13** | 2008 | Subsequent documented reprint | Same photograph retained | Continued use within the same documented **image–condition configuration** | Institutional library record |
| **14** | 2010 | Subsequent documented edition/reprint | Same photograph retained | Continued use within the same documented **image–condition configuration** | Institutional library record |
| **15** | 2015 | Second edition | Same photograph retained in a new edition | The same observable relationship among image location, condition identification by name, declared curricular location of the condition, location of substantive clinical description, and the destination indicated in the accompanying text was retained in the new edition. | Institutional library record |
| **16** | 2015 | Second-edition reprint I | Same photograph reproduced | Same documented **image–condition configuration** retained | Bibliographic reconstruction |
| **17** | 2015 | Second-edition reprint II | Same photograph reproduced | Same documented **image–condition configuration** retained | Bibliographic reconstruction |
| **18** | 2015 | Second-edition reprint III | Same photograph reproduced | Same documented **image–condition configuration** retained | Bibliographic reconstruction |
| **19** | 2015 | Second-edition reprint IV | Same photograph reproduced | Same documented **image–condition configuration** retained | Bibliographic reconstruction |
| **20** | 2015 | Second-edition reprint V | Same photograph reproduced | Same documented **image–condition configuration** retained | Bibliographic reconstruction |
| **21** | 2015 | Second-edition reprint VI | Same photograph reproduced | Same documented **image–condition configuration** retained | Bibliographic reconstruction |
| **22** | 2015 | Second-edition reprint VII | Same photograph reproduced | Same documented **image–condition configuration** retained | Bibliographic reconstruction |
| **23** | 2016 | Second edition, reprint 1 | Same photograph reproduced | Continued use within the same documented **image–condition configuration** | Institutional library record |
| **24** | 2016 | Second edition, reprint 2 | Same photograph reproduced | Continued use within the same documented **image–condition configuration** | Bibliographic reconstruction |
| **25** | 2017 | Second edition, reprint 3 | Same photograph reproduced | Continued use within the same documented **image–condition configuration** | Bibliographic reconstruction |
| **26** | 2017 | Second edition, reprint 4 | Same photograph reproduced | Continued use within the same documented **image–condition configuration** | Bibliographic reconstruction |
| **27** | 2018 | Second edition, reprint 5 | Same photograph reproduced | Continued use within the same documented **image–condition configuration** | Institutional library record |
| **28** | 2019 | Second edition, reprint 6 | Same photograph reproduced | The same documented **image–condition configuration** remained present 14 years after the photograph’s first inclusion in the analyzed textbook lineage. | Physical copy / publication imprint |

### Explanatory Note to Table S1

In this table, **image–condition configuration** is used descriptively to refer to the observable relationship among **the clinical photograph, the clinical condition identified by name at the point of image presentation, the condition’s declared curricular location within the textbook structure, the location of its substantive clinical description, and the destination indicated in the text accompanying the photograph**. The term does not attribute an educational intention, learning objective, or rationale for image placement to the authors, editors, or publisher.

Persistence of the same **image–condition configuration** across successive editions and reprints does **not**, by itself, establish that the photograph lacked educational value, that its placement was inappropriate, or that its educational justification diminished over time. The **28 documented editions and reprints** are used here as a longitudinal sequence through which persistence of the observable configuration can be examined.

The evidence categories identify the documentary sources through which individual editions or reprints were identified or verified. Sources used in the reconstruction included **physical copies and publication imprints, institutional library holdings or catalogue records, online antiquarian or second-hand bibliographic records, online marketplace bibliographic records, and bibliographic reconstruction from multiple documentary sources**. Individual editions and reprints were not necessarily identified or verified through the same source type, and the evidence designation should **not be interpreted as indicating that every item was physically examined**.

Where more than one documentary source was available for the same edition or reprint, the available sources were compared as part of the bibliographic reconstruction. **Full bibliographic identifiers and supporting documentary material are not reproduced in this public Appendix where their inclusion could unnecessarily facilitate retrieval of the underlying highly sensitive pediatric clinical photographs. They may be provided to the editors for verification.**

## **Table S2.** Subsequent reassessment and curricular persistence events, 2023-2026.

| **Phase** | **Year** | **Documented event** | **Educational / institutional context** | **Status of image or material** | **Analytical significance** | **Evidence** |
| --- | --- | --- | --- | --- | --- | --- |
| **Discovery and objection** | 2023–2025 | Discovery of continued circulation followed by formal objection by the person depicted | Post-publication educational environment | The person depicted became aware of the continued use and objected to further use of the photograph. | New information entered the educational life cycle of the image, creating a documented trigger for reassessment; **this was not a new publication event**. | Documented objection and correspondence |
| **Authorization status and documentation inquiry** | 2025 | Publisher response to an institutional inquiry concerning consent documentation | Scientific publisher / institutional inquiry | **The person depicted reports that she did not consent to the educational publication or repeated reuse of the photographs. Separately, the publisher stated that it did not possess the requested consent documentation and described reliance on contractual declarations made by authors.** | **Two distinct findings concerning authorization status and the reconstructability of consent documentation became available for contemporary reassessment.** | Statement of the person depicted; documented objection and correspondence; publisher correspondence, May 12, 2025 |
| **Institutional reassessment** | 2025 | Withdrawal of relevant textbook holdings from the university library collection and catalogue | Pomeranian Medical University in Szczecin | Relevant editions and reprints were removed; **146 copies were withdrawn in total**. | Documented example of institutional reassessment followed by modification of access to existing educational material. | University library documentation, October 6, 2025 |
| **Institutional reassessment** | 2026 | Review and amendment of teaching syllabi | Pomeranian Medical University in Szczecin | Identified textbooks were removed from relevant recommended-reading lists following institutional review. | Documented example of reassessment leading to modification of formal curricular recommendations. | Rector’s correspondence, February 2, 2026 |
| **Post-objection curricular persistence** | 2026 | Documented continued curricular use of existing editions in other higher-education curricula | Medical and health-professions education | Existing editions from the analyzed textbook lineage remained designated, depending on the institution, as basic, mandatory, recommended, or supplementary literature. |  |  |

**The events presented in Table S2 are analytically distinct from the 28 documented editions and reprints listed in Table S1.** They concern material already in circulation and are therefore not counted as additional publication or reproduction events. Their relevance lies in documenting subsequent changes in the information available to responsible entities, institutional reassessment, and continued curricular use of previously published material.

## Definition of the Image–Condition Configuration

For the purposes of this reconstruction, **image–condition configuration** refers to the observable relationship among five components of the educational material:

1. the **clinical photograph**;
2. the **clinical condition identified by name at the point of image presentation**;
3. the condition’s **declared curricular location** within the textbook structure;
4. the location of its **substantive clinical description**; and
5. the **destination indicated in the text accompanying the photograph**.

Across the **28 documented editions and reprints of the analyzed textbook lineage between 2005 and 2019**, these elements did not converge on the same location.

**At the point of image presentation, the depicted clinical condition was identified by name, but no substantive clinical description of that condition was provided there.** The condition was not identified in the textbook’s table of contents as a component of the chapter containing the photograph. Instead, the text accompanying the photograph stated that the condition was described elsewhere in the textbook, and its substantive clinical description was located at that other curricular location. **The highly sensitive pediatric clinical photograph itself remained in a different location.**

This configuration does **not** demonstrate that the photograph lacked educational value, that its placement was inappropriate, or that repeated use was itself problematic. Nor does it permit inference about the intentions, motives, learning objectives, or educational reasoning of the authors, editors, or publisher.

Rather, it documents an observable structural relationship that gives rise to the central educational question developed in the main manuscript:

**What additional educational information does the photograph contribute at the point of its actual presentation?**

If the photograph provides a distinct context-specific contribution—for example, by enabling recognition of a phenotype, distribution of clinical features, anatomical proportions, diagnostic differentiation, or another clinically relevant relationship—its placement and continued use may remain educationally justified. The reconstruction itself does not predetermine the outcome of that assessment.

The significance of the configuration is therefore not that the photograph and the substantive clinical description of the depicted condition are physically separated. **Physical separation and cross-referencing may be educationally appropriate.** Its analytical significance lies in requiring a distinction among **identification by name of the condition depicted with the photograph, identification of where that condition is substantively described, and identification of what additional educational information the photograph itself contributes at the point of presentation**.

## Textbook Publication Persistence and Curricular Persistence

The longitudinal reconstruction distinguishes between **two analytically different forms of persistence**.

**Textbook publication persistence** refers to repeated inclusion of the same photograph and persistence of the documented **image–condition configuration** across the **28 identified editions and reprints of a single academic textbook lineage between 2005 and 2019**.

This form of persistence establishes that the photograph and the observable relationship among **image location, condition identification by name, declared curricular location of the condition, location of substantive clinical description, and the destination indicated in the accompanying text** remained present across the reconstructed textbook sequence. It does **not**, by itself, establish loss of educational value or weakening of educational justification.

**Curricular persistence** refers to continued formal incorporation of previously published editions into contemporary educational programs after the last identified reprint in the analyzed 2005–2019 sequence.

This distinction is important because the presence of an existing textbook in a contemporary curriculum does not constitute a new edition, reprint, or reproduction of the photograph. It demonstrates instead that **previously published material continues to function as a formally designated educational resource**.

Institutional documentation confirms that editions from the analyzed textbook lineage remained incorporated into educational programs after the 2005–2019 publication sequence ended. Documentation for the 2025/2026 academic year identifies an existing edition from the analyzed textbook lineage as basic course literature. Documentation for the **2026/2027 academic year** again designates an existing edition from that lineage as basic course literature.

Other institutional curricular documentation demonstrates additional forms of formal educational use of editions from the same textbook lineage, including designation as mandatory, basic, recommended, or supplementary literature according to the respective curriculum.

These observations are not used to infer how individual learners accessed the textbook, whether they viewed particular photographs, or how those photographs affected learning. Nor do they establish that every copy used in every course contained an identical image–condition configuration unless independently verified. Their narrower evidentiary function is to establish the **continued formal curricular status of previously published editions from the analyzed textbook lineage**.

Accordingly, curricular documentation from 2026 is **not counted among the 28 editions and reprints** and does not constitute an additional publication event. It represents a subsequent phase in the educational life cycle of previously published material.

The distinction can therefore be summarized as follows:

**Textbook publication persistence** concerns the continued presence of the photograph and its documented **image–condition configuration** across successive editions and reprints.

**Curricular persistence** concerns the continued formal educational use of those previously published materials after the publication sequence itself has ended.

## Formal Objection as a Reassessment Trigger

The longitudinal trajectory contains an event fundamentally different from publication, reproduction, or curricular inclusion: **formal objection by the person depicted in the photograph**.

The person depicted discovered the continued circulation of the photograph in 2023 and subsequently objected to its further use during 2023–2025.

For purposes of the present analysis, this objection is not used to retrospectively determine whether individual historical publications were lawful or ethically permissible. Nor does the objection itself establish that the photograph lacks educational value or that continued use must cease.

Its analytical significance is different: **the objection represents new information entering the educational life cycle of an already circulating highly sensitive clinical image.**

This distinction is important for longitudinal educational governance. Reassessment need not be triggered solely by elapsed time, creation of a new edition, or scheduled revision of an educational resource. It may also be triggered by a **material change in the information available to the entity responsible for continued use**.

A documented objection by the person depicted is one such event.

Once such information becomes available, the informational conditions under which continued use is evaluated have changed. The responsible entity can then reassess, among other considerations:

- the image’s current educational function;
- its image-specific educational contribution at the point of presentation;
- its relationship to the **image–condition configuration**;
- the proportionality between educational function and scope of patient exposure;
- the availability of equivalent alternatives;
- the current conditions of consent, assent, objection, or other relevant authorization;
- identifiability and risks associated with contemporary circulation; and
- the conditions under which continued use could remain educationally and ethically justified.

The presence of an objection does **not** predetermine the outcome of this reassessment. It does not establish that the photograph has lost educational value, nor does it automatically require withdrawal. **It changes the information available to the responsible entity and thereby creates a reason to reassess the conditions and justification for continued use.**

This distinction is central to the **event-triggered logic of the Educational Reassessment Framework** proposed in the main manuscript.

## Authorization Status and Reconstructability of Documentation as Part of Reassessment

The longitudinal reconstruction provides **two analytically distinct sources of information concerning authorization**.

First, **the person depicted, who is also the author of this Viewpoint, reports that she did not provide consent for the educational publication or repeated reuse of these childhood photographs**. This concerns the reported authorization status from the perspective of the person whose images were used.

Second, the 2025 institutional inquiry provided information concerning **the contemporary reconstructability of documentation supporting use**. In correspondence dated May 12, 2025, the publisher stated that it did **not possess the requested consents concerning the photographed persons** and explained that its publication arrangements relied on contractual declarations made by authors concerning, among other matters, copyright, personal data, and the legality of materials supplied for publication. The publisher further stated that it had not independently approached photographed persons or otherwise verified author-held consent beyond such declarations.

These two observations should not be collapsed into a single evidentiary inference. **The reported absence of consent by the person depicted and the publisher’s inability to produce the requested consent documentation are analytically distinct findings.** The first concerns the reported authorization status of the person depicted; the second concerns the contemporary reconstructability of documentation supporting use.

**Educational value and authorization are analytically distinct dimensions.** A photograph may retain substantial educational value even when the conditions supporting its use require reassessment. Conversely, authorization does not itself establish why a particular photograph is educationally necessary at a particular location. The question of **whether the image may appropriately be used** and the question of **what educational contribution the image provides here** therefore remain distinct.

For purposes of longitudinal reassessment, these findings introduce different but related information into the educational life cycle of the image:

- **the statement of the person depicted provides information concerning her reported authorization status and position regarding continued use;**
- **the publisher’s response provides information concerning the contemporary reconstructability of documentation supporting that use.**

Neither finding determines the image’s educational value. Both are relevant to assessing the conditions under which continued use may be considered responsible.

This distinction supports the event-triggered structure proposed in the main manuscript: **reassessment is prompted not simply by the passage of time or repeated reproduction, but when a material event changes the educational context, the conditions of use, or the information available for evaluating continued use.**

The longitudinal sequence can therefore be understood as moving through analytically distinct stages:

**publication persistence → new material information → reassessment → documented institutional decision → continued or modified curricular use.**

These stages should not be collapsed into a single measure of continued use. Their significance lies in showing how a clinical image may remain materially unchanged while **the educational context, available information, and conditions under which continued use is evaluated change over time**.
